# Physical Activity and Sedentary Behaviour Patterns among Kenyan and Japanese Children: A Comprehensive Cross-Country Comparison

**DOI:** 10.3390/ijerph17124254

**Published:** 2020-06-15

**Authors:** Tetsuhiro Kidokoro, Noriyuki Fuku, Toshio Yanagiya, Tomonari Takeshita, Mizuki Takaragawa, Michael Annear, Tian Xiaojie, Luka B. Waiganjo, Lamec F. Bogonko, Juliet K. Isika, Mbithe D. Kigaru, Francis M. Mwangi

**Affiliations:** 1 Physical Fitness Research Institute, Meiji Yasuda Life Foundation of Health and Welfare, Tokyo 192-0001, Japan; 2 Department of Health & Physical, Education College of Arts & Science, International Christian University, Tokyo 181-8585, Japan; 3 Graduate School of Health and Sports Science, Juntendo University, Chiba 270-1695, Japan; noriyuki.fuku@nifty.com (N.F.); tyanagi@juntendo.ac.jp (T.Y.); sh4219011@juntendo.ac.jp (T.T.); sh4219010@juntendo.ac.jp (M.T.); 4 Faculty of Sport Sciences, Waseda University, Tokyo 202-0021, Japan; annear@aoni.waseda.jp; 5 Faculty of Health and Sport Sciences, University of Tsukuba, Ibaraki 305-8574, Japan; tian.xiaojie.fw@u.tsukuba.ac.jp; 6 Department of Physical Education, Exercise & Sports Science, Kenyatta University, Nairobi, P.O. Box 43844-0010, Kenya; waiganjo.luka@ku.ac.ke (L.B.W.); lamech.bogonko@ku.ac.ke (L.F.B.); mwangi.francis@ku.ac.ke (F.M.M.); 7 Department of Fashion Design and Marketing, Kenyatta University, Nairobi, P.O. Box 43844-0010, Kenya; isika.juliet@ku.ac.ke; 8 Department of Food, Nutrition and Dietetics, Kenyatta University, Nairobi, P.O. Box 43844-0010, Kenya; kigaru.dorcus@ku.ac.ke

**Keywords:** exercise, screen time, Africa, Asia, accelerometers

## Abstract

Health benefits of physical activity are well known, yet available physical activity data is limited from children living in African and Asian countries. The purpose of the cross-sectional study was to evaluate and compare physical activity and sedentary behavior patterns, particularly hourly variations, among children in Kenya and Japan. Participants included 298 primary school students (122 Kenyan, 176 Japanese) aged 9–12 years. Physical activity and sedentary behavior were measured with accelerometers. Domain-specific physical activity, screen time, and proportion of children using active transport to school were measured by questionnaire. A two-way ANOVA (countries × time) was used to examine the differences in the activity patterns between Kenyan and Japanese children. The results from the present study demonstrated that Kenyan children spent more time in moderate-to-vigorous physical activity compared to Japanese children (*p* < 0.05) with the greatest differences found for weekday evenings (for boys and girls) and weekend afternoons (for girls). This suggests that these were ‘critical periods’ to differentiate the physical activity levels between Kenyan and Japanese children. However, a higher proportion of the children from Japan used active transport to school and spent less time in television viewing and computer gaming. The results suggest that both countries have successes and challenges that can aid in developing effective and country-specific intervention strategies for promoting physical activity.

## 1. Introduction

The benefits of regular physical activity among school-aged children are well documented and include positive effects on physical, physiological, developmental, mental, cognitive, and social health, as well as academic achievement [1,2]. Despite these benefits, it has been suggested that the majority of school-aged children worldwide do not meet physical activity guidelines [3,4] requiring children to engage in at least 60 min of moderate-to-vigorous physical activity (MVPA) every day [5]. Additionally, a cross-country comparison study has shown great variability in physical activity across countries and continents, with physical activity often measurably lower in high-income countries [4]. The reduced physical activity in high-income countries is referred to as the ‘physical activity transition’ resulting from widespread behavioral shifts to more sedentary lifestyles [6]. As a result, prolonged sedentary behavior has become a global problem in modern societies, including Japan, and has been shown to adversely affect cardiometabolic risk markers [7]. Importantly, there is often little association between physical activity and sedentary behavior, suggesting that physical activity and sedentary behavior are independently associated with cardiometabolic risk markers in children [8]. Despite the independent benefits of reducing sedentary behavior, time spent in screen-related activities such as television viewing, computer gaming, and using smartphones are now highly present among children. Indeed, many children in developed countries fail to achieve screen time recommendation suggesting children engage in less than 2 h of screen time per day [9,10]. 

To better understand how economic development and urbanization influence humans’ activity patterns, findings from cross-country comparison studies with diverse socio-cultural backgrounds can be useful. However, most available evidence comes from studies conducted in European and North American countries. Data are limited from Asian and African countries [4,11].

Sub-Saharan Africa, including Kenya, is currently undergoing rapid social, cultural, economic, and urban development. Alongside these changes, serious public health problems have emerged including declines in habitual physical activity (e.g., traditional practices of walking long distances and manual labor), increases in sedentary behavior (e.g., using motorized transport and increased screen time), and subsequent increases in childhood obesity [12,13,14]. Similarly, evidence shows substantial declines in physical fitness of Asian children [15], warranting prompt action among these populations. According to the United Nations, most of the world’s population growth over the next few decades is projected to occur in Asia and Africa [16]. Therefore, it is essential to better understand effective interventions to fight against further downward trends in physical activity in those regions. 

Although previous cross-country comparison studies evaluating physical activity levels among children including both developed and developing countries have provided useful insights [3,4], those studies predominantly used questionnaires to evaluate physical activity levels. This limits accuracy and reliability of the data; this is particularly the case in pediatric populations [17]. Recently, wearable sensors such as pedometers and accelerometers have been widely used in physical activity research, which can provide more accurate and reliable data. A previous study evaluating step counts among Kenyan children aged 9–11 years old using pedometers demonstrated that children in living in rural areas were more active than children in urban areas [14]. However, it is not known how Kenyan children spent their time across a day from the perspective of physical activity (i.e., hourly variations in physical activity). Understanding the detail of physical activity patterns among Kenyan children will help us to clarify ‘when’ they are active (or inactive), which can be a useful insight to implement domain-specific interventions among those populations. 

Furthermore, evidence has shown the importance of active school transport such as walking to school to increase physical activity in children. Indeed, a previous systematic review showed that children who walk to school are more likely to meet physical activity guidelines than children who use motorized transport [18]. However, it was suggested that there were great differences in prevalence of active school transport across countries, suggesting that those habits are more likely to be influenced by environmental and sociological factors [19]. 

The main purpose of the present study was to evaluate and compare physical activity and sedentary behavior patterns, focusing particularly on hourly variations in activity among Kenyan children in comparison to Japanese children. Additionally, the present study aimed to compare prevalence of active school transport as well as screen time among Kenyan and Japanese children.

## 2. Materials and Methods

### 2.1. Participants

The present study was a cross-sectional study comparing physical activity and sedentary behavior patterns between Kenyan and Japanese children. This study employed a purposive sample of one primary school from both Kenya (Nairobi Province) and Japan (Nagano prefecture). Schools were selected based on the following criteria, which provided a basis for comparison: providing elementary level education as part of a national curriculum, location in peri-urban areas. School children aged 9 to 12 years from the two aforementioned schools participated in the study. A total of 327 activity logs were collected in Kenya (*n* = 145) and Japan (*n* = 182). Measurements were conducted in June–July 2019 in both countries. Parents/guardians provided written, informed consent for their children to participate in the study. The study was conducted in accordance with the Declaration of Helsinki and approved by the institutional ethical advisory committee of International Christian University (project identification code: 2019-25) and Kenyatta University (project identification code: PKU/955/11045).

### 2.2. Measurements

#### 2.2.1. Anthropometry Assessments

Bodyweight was measured using a digital scale, accurate to the nearest 0.1 kg, and height was measured using a stadiometer, accurate to the nearest 0.1 cm. Body mass index (BMI) was calculated as weight (kg) divided by the square of the height (m^2^).

#### 2.2.2. Physical Activity and Sedentary Behavior with Accelerometers

Physical activity and sedentary behavior were measured by three-axis accelerometers (ActiGraph wGT3X-BT, LLC, Pensacola, FL, USA). The accelerometers have been shown to be valid and reliable activity monitors for measuring physical activity and sedentary behavior in children [20,21]. The participants were asked to wear the accelerometer on the right side of their hip using a belt for seven consecutive school days (Monday to Sunday) except when sleeping or during water-based activities (e.g., showering or swimming). Data were collected in 15 s epochs. Non-wear time was defined as a period of ≥ 60 min of continuous zero counts as recorded on the ActiGraph [22]. Only the participants with ≥ 10 h of wear time per day for a minimum of four days (including at least one weekend day) were included in the analyses [23]. Evenson’s cut-off points [20] were used to categorize the activities into three levels: sedentary behavior, < 101 counts per min (CPM); light-intensity physical activity (LPA), 101–2295 CPM; moderate-intensity physical activity (MPA), 2296–4011 CPM; vigorous-intensity physical activity (VPA), ≥ 4012 CPM; MVPA, ≥ 2296 CPM. The collected data were analyzed using the ActiLife software, version 6.13.3 (ActiGraph, LLC, Pensacola, FL, USA).

#### 2.2.3. Domain-Specific Physical Activity

Domain-specific physical activity was assessed by using The Physical Activity Questionnaire for Older Children (PAQ-C) [24,25]. The PAQ-C is a self-administered, 7-day recall instrument developed particularly for children aged 8–14 years old. Questionnaire items include weekly effort during physical education and activity during lunch, after school, evenings, and weekends. Evidence was provided that supported the PAQ-C as a reliable and valid measure of physical activity levels in children [24,25]. Children were asked to answer their physical activity levels in each domain using a five-point scale (scoring for each item from 1–5) [24,25]. The lowest activity response was evaluated as 1 and the highest activity response was evaluated as 5 (i.e., higher scores indicate higher physical activity levels) [24,25]. Additionally, children were asked to respond to the following question: ‘Do you belong to an after-school sport club?’ with answering ‘yes (coded as 1)’ or ‘no (coded as 0)’. 

#### 2.2.4. Screen Time

Screen time was assessed with a lifestyle questionnaire from the International Study of Childhood Obesity, Lifestyle and the Environment (ISCOLE) study [26]. Participants were asked about the amount of time spent watching television or playing video games for both weekdays and weekend. 

#### 2.2.5. Active School Transport

Active school transport was assessed by a questionnaire that was used in previous international comparison studies [19,27]. The participants were asked about the main mode of transport that they used to go to school during the last week. The response options included physically active modes such as walking, bicycling; and non-active modes such as car, motorcycle, bus, train. We classified participant’s mode of transport into two categories, ‘active school transport’ versus ‘non-active school transport’ based on the above categories. The participants were also asked about the time spent during the journey from home to school. The response options were: < 5 min, 5–15 min, 16–30 min, 31 min–1 h and >1 h. For the analysis, the participants were categorized into two groups (i.e., ‘0–30 min’ and ‘>30 min’).

### 2.3. Statistical Analysis

It has been suggested that there are significant differences in physical activity and sedentary behavior between boys and girls [28], therefore, all analyses were conducted by gender. To examine any differences between the Kenyan and Japanese children’s basic characteristics, physical activity, and sedentary behavior, independent t-tests were performed. A Mann–Whitney U test was used to examine differences between the two countries in the proportion of children participating in after-school sport activities. A Mann–Whitney U test was also used to examine the differences between Kenyan and Japanese children in the proportion of children using active transport to school. 

Physical activity and sedentary behavior patterns were ascertained by calculating the time spent in each activity per hour. For example, activity at 7:00 a.m. was ascertained by calculating the time spent in activities between 6:30 a.m. and 7:30 a.m. [29]. At the participating school in Kenya, the students were at the institution from 8:00 a.m. to 4:30 p.m. (normal Kenyan public-school hours), which included three breaks (9.30 a.m. to 9.50 a.m., 11.00 a.m. to 11.30 a.m., and 12:40 p.m. to 2:00 p.m.). In contrast, at the participating school in Japan, the students were at the institution from 8:20 a.m. to 4:00 p.m. (normal Japanese public-school hours), which included three breaks (10:40 a.m. to 11:00 a.m., 12:30 p.m. to 1:50 p.m., and 2:50 p.m. to 3:05 p.m.). A two-way ANOVA (counties × time) was used to examine the differences in the activity patterns between Kenyan and Japanese children. When a significant difference was observed in the two-way ANOVA (counties × time) analyses, a simple main effect (country) at a given time point was ascertained to examine the difference in activities between Kenya and Japan. Statistical analyses were conducted using SPSS version 24 (SPSS, Inc., IBM, Armonk, NY, USA).

## 3. Results

### 3.1. Basic Characteristics of the Participants, Objectively Measured Physical Activity, and Sedentary Behavior

Among 327 children (145 Kenyan and 182 Japanese) from whom written informed consent was obtained from their parents or guardians, 29 participants (23 Kenyan and 6 Japanese) who did not provide valid data were excluded from the final analysis. The final sample for the present study, therefore, comprised 298 children (122 Kenyan and 176 Japanese) aged 9–12 years (valid data = 91.1%). Table 1 shows the basic characteristics of the participants, objectively measured physical activity, and sedentary behavior. For weekday activity, Kenyan boys had higher step counts and spent more time in MPA than Japanese boys (*p* < 0.005). In addition, Kenyan girls spent more time in MVPA, MPA, VPA and had higher step counts than Japanese girls (*p* < 0.05). For weekend activity, there was no significant difference in MVPA or step counts between Kenyan and Japanese boys (*p* > 0.100). However, there were significant differences in weekend MVPA, MPA, and VPA between Kenyan and Japanese girls (*p* < 0.001). 

### 3.2. Weekday Physical Activity and Sedentary Behavior Patterns across a Day among Kenyan and Japanese Boys

**Figure 1:**
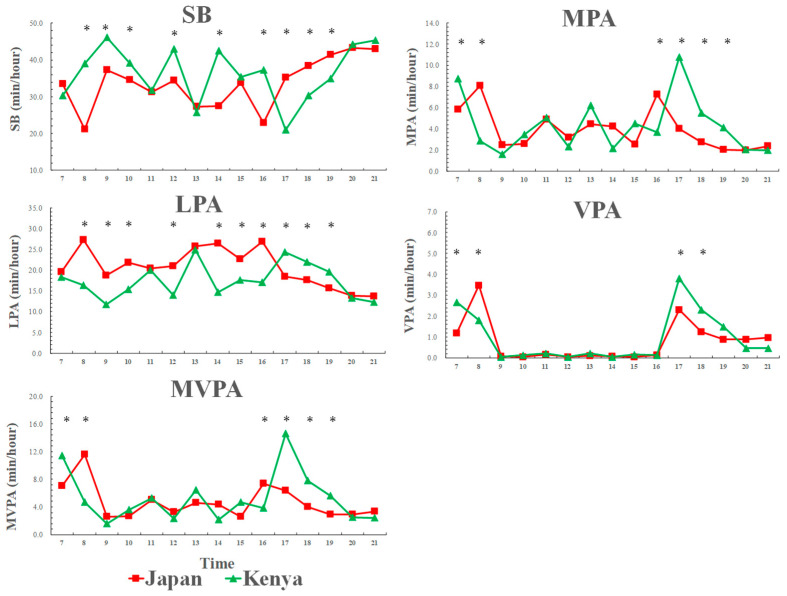
Weekday physical activity and sedentary behavior patterns across a day among Kenyan and Japanese boys. SB: sedentary behavior; LPA: light-intensity physical activity; MPA: moderate-intensity physical activity; VPA: vigorous-intensity physical activity; MVPA: moderate-to-vigorous-intensity physical activity. SB [country: F = 7.1, *p* = 0.009; time: F = 23.5, *p* < 0.001; country × time: F = 13.2, *p* < 0.001], LPA [country: F = 11.5, *p* = 0.001; time: F = 21.0, *p* < 0.001; country × time: F = 15.5, *p* < 0.001], MPA [country: F = 3.9, *p* = 0.051; time: F = 6.2, *p* < 0.001; country × time: F = 2.9, *p* < 0.001], VPA [country: F = 1.1, *p* = 0.284; time: F = 8.0, *p* < 0.001; country × time: F = 2.0, *p* = 0.045], MVPA [country: F = 0.8, *p* = 0.370; time: F = 29.5, *p* < 0.001; country × time: F = 14.6, *p* < 0.001]; * significant difference between Kenyan and Japanese children, *p* < 0.05.

Figure 1 shows that weekday physical activity and sedentary behavior patterns across a day among Kenyan and Japanese boys. From morning to evening (7:00–16:00), Japanese boys spent less time in sedentary behavior and more time in LPA compared to Kenyan boys. However, in the evening time (17:00–19:00), Kenyan boys spent less time in sedentary behavior and more time in LPA compared to Japanese boys. Additionally, Kenyan boys spent more time in MVPA in the evening time (17:00–19:00) than Japanese boys.

### 3.3. Weekday Physical Activity and Sedentary Behavior Patterns across a Day among Kenyan and Japanese Girls

**Figure 2:**
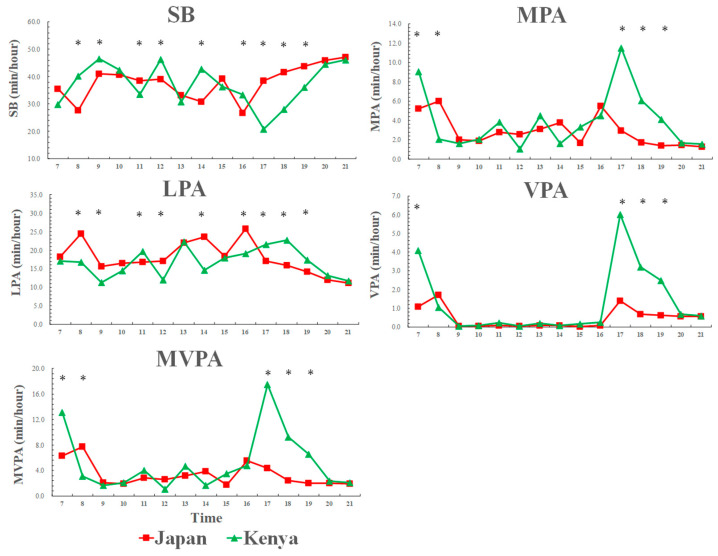
Weekday physical activity and sedentary behavior patterns across a day among Kenyan and Japanese girls. SB: sedentary behavior; LPA: light-intensity physical activity; MPA: moderate-intensity physical activity; VPA: vigorous-intensity physical activity; MVPA: moderate-to-vigorous-intensity physical activity. SB [country: F = 5.1, *p* = 0.026; time: F = 22.9, *p* < 0.001; country × time: F = 17.3, *p* < 0.001], LPA [country: F = 3.1, *p* = 0.080; time: F = 21.0, *p* < 0.001; country × time: F = 10.2, *p* < 0.001], MPA [country: F = 1.6, *p* = 0.208; time: F = 17.3, *p* < 0.001; country × time: F = 13.2, *p* < 0.001], VPA [country: F = 6.8, *p* = 0.010; time: F = 19.4, *p* < 0.001; country × time: F = 5.4, *p* < 0.001], MVPA [country: F = 33.1, *p* < 0.001; time: F = 52.7, *p* < 0.001; country × time: F = 34.3, *p* < 0.001]; * significant difference between Kenyan and Japanese children, *p* < 0.05.

Figure 2 shows that weekday physical activity and sedentary behavior patterns across a day among Kenyan and Japanese girls. Similar to the boy’s results, from morning to evening (7:00–16:00), Japanese girls had less time in sedentary behavior and more time in LPA compared to Kenyan girls. However, at evening time (17:00–19:00), Kenyan girls spent less time in sedentary and more time in LPA compared to Japanese girls. Kenyan girls also spent more time in MVPA at the evening time (17:00–19:00) than Japanese girls.

### 3.4. Weekend Physical Activity and Sedentary Behavior Patterns across a Day among Kenyan and Japanese Boys

**Figure 3:**
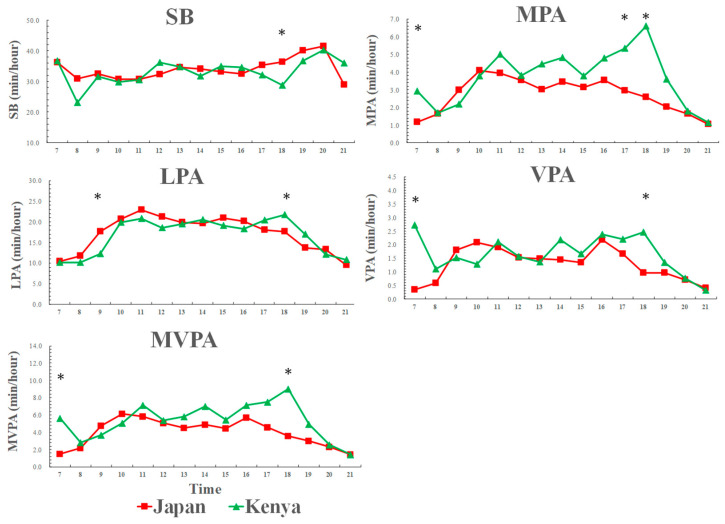
Weekend physical activity and sedentary behavior patterns across a day among Kenyan and Japanese boys. SB: sedentary behavior; LPA: light-intensity physical activity; MPA: moderate-intensity physical activity; VPA: vigorous-intensity physical activity; MVPA: moderate-to-vigorous-intensity physical activity. SB [country: F = 0.6, *p* = 0.432; time: F = 2.1, *p* = 0.010; country × time: F = 1.6, *p* = 0.047], LPA [country: F = 0.3, *p* = 0.576; time: F = 12.5, *p* < 0.001; country × time: F = 2.7, *p* = 0.001]. MPA [country: F = 2.7, *p* = 0.111; time: F = 6.4, *p* < 0.001; country × time: F = 2.8, *p* < 0.001], VPA [country: F = 1.7, *p* = 0.208; time: F = 3.1, *p* < 0.001; country × time: F = 1.7, *p* = 0.048], MVPA [country: F = 2.4, *p* = 0.128; time: F = 5.6, *p* < 0.001; country × time: F = 2.5, *p* = 0.002]; * significant difference between Kenyan and Japanese children, *p* < 0.05.

Figure 3 shows that weekend physical activity and sedentary behavior patterns across a day among Kenyan and Japanese boys. There was only one-time segment (at 18:00) when the significant difference in sedentary behavior was found between the two countries. Kenyan boys spent less time in sedentary behavior and more time in LPA than Japanese boys during the weekend evenings. In addition, Kenyan boys spent more time in MVPA than Japanese boys at 18:00.

### 3.5. Weekend Physical Activity and Sedentary Behavior Patterns across a Day among Kenyan and Japanese Girls

**Figure 4:**
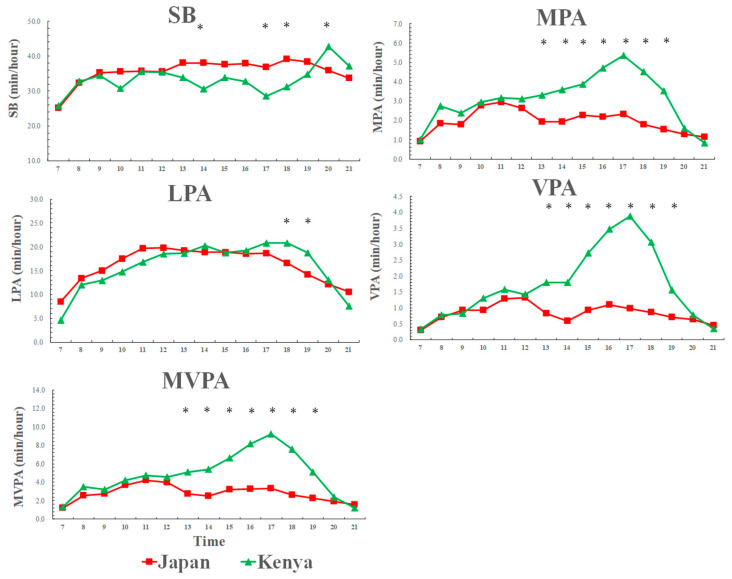
Weekend physical activity and sedentary behavior patterns across a day among Kenyan and Japanese girls. SB: sedentary behavior; LPA: light-intensity physical activity; MPA: moderate-intensity physical activity; VPA: vigorous-intensity physical activity; MVPA: moderate-to-vigorous-intensity physical activity. SB [country: F = 3.1, *p* = 0.089; time: F = 2.1, *p* = 0.009; country × time: F = 1.6, *p* = 0.047], LPA [country: F = 0.1, *p* = 0.913; time: F = 11.7, *p* < 0.001; country × time: F = 1.8, *p* = 0.040], MPA [country: F = 8.0, *p* = 0.008; time: F = 4.6, *p* < 0.001; country × time: F = 2.0, *p* = 0.015], VPA [country: F = 18.1, *p* < 0.001; time: F = 7.4, *p* < 0.001; country × time: F = 5.0, *p* < 0.001], MVPA [country: F = 14.3, *p* = 0.001; time: F = 6.6, *p* < 0.001; country × time: F = 3.7, *p* < 0.001]; * significant difference between Kenyan and Japanese children, *p* < 0.05.

Figure 4 shows that weekend physical activity and sedentary behavior patterns across a day among Kenyan and Japanese girls. The significant differences in activity patterns were found in the weekend afternoons: Kenyan girls spent less time in sedentary behavior and more time in LPA compared to Japanese girls in the afternoons. Particularly, significant differences in MVPA were found in the weekend afternoon to evening times (from 13:00–18:00) between Kenyan and Japanese girls.

### 3.6. Domain-Specific Physical Activity and Screen Time among Kenyan and Japanese Children

Table 2 shows domain-specific physical activity and screen time among Kenyan and Japanese children. Japanese children (both boys and girls) had higher physical activity scores during ‘physical education classes’ than Kenyan children (*p* < 0.001). Japanese boys also had a higher proportion of children participating in after-school sport activities than that of Kenyan boys (*p* = 0.009). Kenyan girls demonstrated higher physical activity scores at ‘right after school’, ‘evening’, and ‘weekend’ than Japanese girls (*p* < 0.05). For screen time, Kenyan boys spent more weekend television time than Japanese boys (*p* < 0.001). For girls, Kenyans spent more time in television viewing and computer gaming (both weekdays and weekends) than Japanese girls (*p* < 0.05).

### 3.7. Proportion of Children Using Active Transport to School among Kenyan and Japanese Children

**Figure 5:**
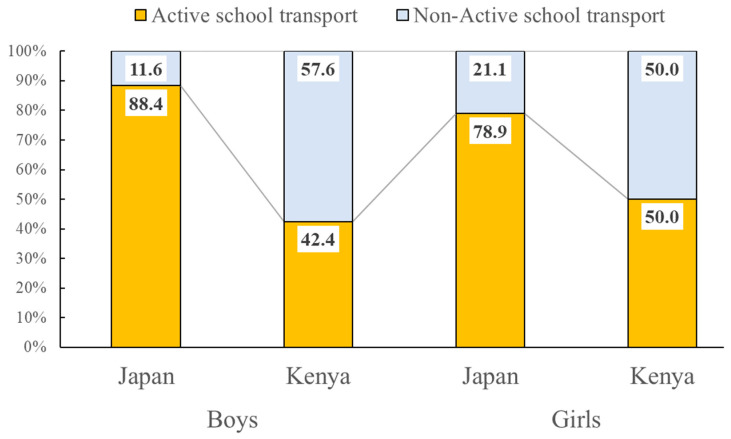
Proportion of children using active transport to school among Kenyan and Japanese children. A Mann–Whitney U test was used to examine the difference in proportion of children using active transport to school among Kenyan and Japanese children. Boys: Mann–Whitney U = 685, *p* < 0.001; girls: Mann–Whitney U = 864, *p* = 0.004.

Figure 5 shows the proportion of children using active transport to school among Kenyan and Japanese children. Japan had a significantly higher proportion of children using active school transport than Kenyan children (*p* < 0.05); 88.4% of Japanese boys used active modes to go to school while 42.4% of Kenyan boys did so. Likewise, 78.9% of Japanese girls used active modes to go to school while 50.0% of Kenyan girls did so.

### 3.8. Duration of School Transport among Kenyan and Japanese Children

**Figure 6:**
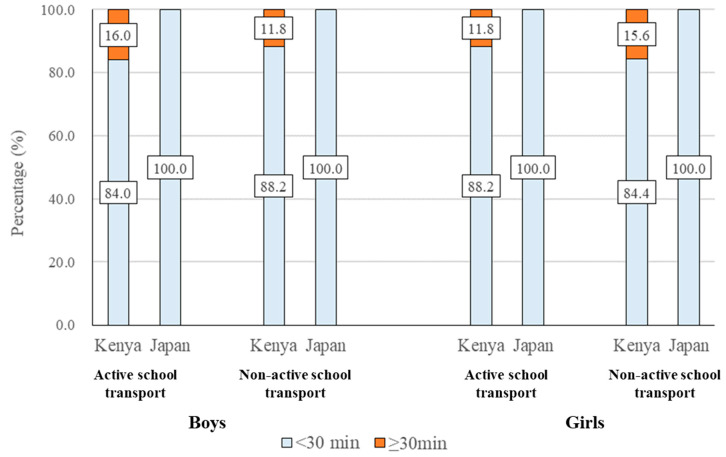
Duration of school transport among Kenyan and Japanese children. The Mann–Whitney U test was used to examine the difference in the duration of school transport among Kenyan and Japanese children. Active school transport, boys: Mann–Whitney U = 399, *p* = 0.011; non-active school transport, boys: Mann–Whitney U = 75, *p* = 0.424; active school transport, girls: Mann–Whitney U = 375, *p* = 0.007; non-active school transport, girls: Mann–Whitney U = 108, *p* = 0.238.

Figure 6 shows the duration of school transport among Kenyan and Japanese children. Among children using active school transport, the proportion of children spending more than 30 min in the school journey was significantly higher for Kenyan children than Japanese children (both for boys and girls, *p* < 0.05). There was no significant difference in the duration of school transport among children using non-active school transport between Kenya and Japan.

## 4. Discussion

### 4.1. Main Findings of the Present Study

The present study demonstrated that Kenyan children spent more time in MVPA compared to Japanese children, particularly in girls. The greatest differences in MVPA between Kenyan and Japanese children were found in weekday evening (17:00–19:00). Regarding weekends, Kenyan girls spent more time in MVPA from afternoon to evening (13:00–19:00) compared to Japanese girls, which resulted in significant differences in total MVPA between Kenyan and Japanese children. These results suggest that weekday evenings (for boys and girls) and weekend afternoons and evenings (for girls) seem to be ‘critical periods’ to differentiate physical activity levels between Kenyan and Japanese children. Although children in Kenya were more active than Japanese children, Japan had a higher proportion of children using active transport to school compared to Kenyan children and Japanese children spent less time in computer gaming (for both boys and girls) and television viewing (for girls) than Kenyan children. 

### 4.2. Development, Urbanization, and Physical Activity

The findings of the present study concur with other studies that show children in less developed societies have higher levels of physical activity than children from comparatively more developed nations [14,30], which may be explained by the physical activity transition model [6]. It is known that country-level factors, such as per capita income, income inequality, and the human development index (HDI) ratings are associated with physical activity levels among children [31,32,33]. A previous cross-country comparison study demonstrated that low- and middle-HDI countries, including Kenya (HDI = 0.579 in 2019) [34], had preferable levels of physical activity and sedentary behaviors (i.e., higher physical activity and lower sedentary behavior) compared to high- and very high-HDI countries [35], including Japan (HDI = 0.915 in 2019) [34]. Furthermore, a study from Kenya that compared physical activity between urban and rural Kenyan children aged 9–12 years old also confirmed that children living in rural areas were more physically active than children living in urban areas [14]. Paradoxically, results have been reported suggesting that lower levels of structure, strategies, and investments to promote physical activity for children and youth were associated with higher levels of physical activity among children [35,36]. Therefore, it is possible that our investments have been less successful in high-income countries given the results from the previous studies [35,36]. These are important implications, particularly for Sub-Saharan African countries such as Kenya because those countries are currently undergoing rapid socio-cultural changes and urbanization. Such changes bring with them a need to fight against global trends for lower activity associated with higher levels of development and urbanization.

### 4.3. Potential Reason for the Differences in Physical Activity Between Kenya and Japan

It is not currently possible to specify the exact reason for stark differences in MVPA between the two countries during weekday evenings (for both boys and girls) and weekend afternoons and evenings (for girls) due to the observational nature of the study. One of the potential reasons for the difference might be due to uniqueness of Kenyan culture where ‘games and club time’ is set immediately after school (3:30 pm to 4:45 pm) per the basic education regulations [37]. The games and club time are used for self-directed, unstructured physical activities, with only a few children engaging in organized/structured sport activities [37]. Our interview with schoolteachers from the Kenyan research school confirmed that the Kenyan children included in the present study were majorly involved in self-directed/unstructured physical activities immediately after school. The children’s involvement in self-directed/unstructured physical activities continues after leaving school [14,38], especially in the peri-urban areas. This often happens in the residential neighborhood during the period preceding 19.00 h (night) around when most parents come home from work and monitor children with their homework assigned at schools [39]. Additionally, the data from our questionnaire survey demonstrated that physical activity score during these periods (immediately after school and evening) were higher in Kenyan children compared to Japanese children. Furthermore, Kenyan children spent more time in VPA during weekday evenings. In addition to the well-established benefits from MVPA [1,2], the additional benefits of VPA among school-aged children are reported, which may have greater beneficial effects on health outcomes in much less time [40,41]. In contrast, especially in developed countries, it is reported that children today play outside less than their parents did and that play has become more structured [42]. Particularly, in Japan, it was reported that youths who do not participate in after-school sport activities are less likely to meet physical activity guidelines, suggesting that the after-school sports activities are a major contributor to Japanese children’s’ physical activity [43]. More developed societies, including Japan, should revisit and promote healthful activity patterns from early stages of social and economic develop to long-lost lifestyles to enable children to accumulate enough physical activity irrespective of the accessibility of after-school sport activities.

### 4.4. Active School Transport

Although Kenyan children were more active than Japanese children particularly during the evening, the present study also showed that Japan had a significantly higher proportion of children using active transport modes for school journeys compared to Kenyan children. A previous systematic review of 68 studies showed that children who walk to school are more likely to meet physical activity guidelines and to have higher cardiovascular fitness than children who use motorized transport [18]. A previous cross-country comparison study showed that Japan had one of the highest rates of active transport to/from school among 49 countries [35]. In Japan, the law requires that public primary school should be sited with no more than 4km from children’s home, which can minimize the use of cars or other forms of motorized vehicles and promote active travel [44]. Consequently, Japan has a well-established and environmentally supported ‘walking to school practice’ [44], which is in line with the results from the present study (88.4% of boys and 78.9% girls used active transport to school). However, it is also important to note that Kenyan children had higher objectively measured MVPA than Japanese children despite the higher proportion of children using active school transport in Japan. This suggests that active school transport might have only a small contribution to overall MVPA among Japanese children, which might be related to the aforementioned law regarding the school district. In other words, since Japanese children live closer to school, using active school transport does not always lead to greater overall MVPA in Japanese children compared to Kenyan children. Indeed, our survey showed that all Japanese children spent less than 30 min in commuting to school, while some Kenyan children (11.8%–15.6%) spent more than 30 min in commuting to school. 

Regarding active school transport in Kenya, only 42.4% of Kenyan boys and 50.0% of Kenyan girls in our sample used active modes of school transport. This is consistent with a previous study showing that only 44.6% of Kenyan children aged 9–11 years living in Nairobi used active transport to school [19]. Active school transport is less common in urban Kenya (such as Nairobi) compared to rural Kenya [14]. This may be explained by the increasing availability of motorized vehicles in rapidly developing cities including Nairobi which can be explained by the physical activity transition model [6]. Motorized travel may also provide a way to avoid dangers on the route to/from school. It has been suggested that child-friendly social and built environments including traffic safety are significantly associated with active school transport among children [45]. Unfortunately, the African region, including Kenya, has the highest road traffic fatality rate in the world, and it is estimated that 38% of these deaths occur among pedestrians [46,47]. Building pedestrian-friendly environments, therefore, may be a prerequisite to promote active school transport among Kenyan children.

### 4.5. Screen Time

The present study also compared time spent in screen time between Kenyan and Japanese children, revealing that Kenyan children spent more time in television viewing and computer gaming than Japanese children. Importantly, Kenyan children had higher screen time as well as simultaneously higher MVPA, which was somewhat consistent with a previous study suggesting that there is only little association between physical activity and screen time in children [8]. One of the potential explanations for this might be ‘compensating behavior’ explained by ActivityStat hypothesis [48]. Namely, when physical activity is increased (or decreased) in one domain, there will be a compensatory change in another domain to maintain an overall stable level of physical activity [48]. Additionally, it is also possible that Japanese children spent more time in non-screen based sedentary behavior (e.g., studying, reading, or listing to music) than Kenyan children, although we do not have data to support the hypothesis.

### 4.6. Physical Education Between Kenya and Japan

The present study demonstrated that Japanese children (both boys and girls) had higher physical activity scores during ‘physical education classes’ than did Kenyan children. Though it is not possible to specify the exact reason, the differences in attitudes toward physical education between Kenya and Japan might be one potential explanation. In Japan, the Ministry of Education, Culture, Sports, Science and Technology (MEXT) sets educational curriculum guidelines for all Japanese elementary schools including the content of physical education and the number of classes, as well as guidelines for school infrastructure and equipment [49]. Although Kenyan government also had a guideline including the duration and frequency of physical education at elementary schools [50], it is suggested that the time slot of physical education is sometimes used to teach examinable subjects due to pressure on schools to perform well academically [38]. Indeed, a previous study suggested that approximately 13% students did not participate in any physical education classes during the past week [14]. Further studies should be conducted to reveal the reason of the differences in physical activity at physical education classes between the two countries.

### 4.7. Strengths and Limitations

The present study has several strengths that support its originality. It is the first study to evaluate physical activity patterns, particularly focusing on their hourly variations in activity among Kenyan and Japanese children. Our comprehensive analysis evaluating physical activity and sedentary behavior patterns across a day revealed the ‘critical period’ that differentiates MVPA between Kenyan and Japanese children. These results will help us to understand effective, domain-specific interventions to promote physical activity among children in both countries. To date, most available physical activity studies have been from European and North American countries, and data from Asia and Africa are limited [11]. The present can substantially expand the literature by adding valuable evidence from socially, economically, and geographically diverse countries. 

Despite the insights provided in our study, some limitations need to be considered. First, it was not possible to identify the reasons for differences in activity patterns between the countries due to the descriptive and cross-sectional design. Individual behaviors are known to be influenced by multiple factors including intrapersonal, interpersonal, policy, and environmental variables [51]. Therefore, implementing intervention studies after controlling those confounding variables will be required to infer causal relationships between our targeted outcomes; such evidence is particularly required from African and Asian countries. Follow-up qualitative investigations may also reveal more about the varied determinants behind patterns of activity. Secondly, only one primary school from Kenya and one from Japan were included in this study, which limits the generalizability of the results. For example, the research school in Kenya was located next to a major road used by many people driving to work in other institutions around the school and in the city. Therefore, this could have influenced the mode and duration of school transport among Kenyan children. The next step for this project will be to develop multi-site surveys. Thirdly, the present study used a self-reported questionnaire to evaluate screen time; however, the reliability and validity of self-reported screen time are mixed and highly variable in children [52]. These results, therefore, should be interpreted with caution. Fourthly, the present study did not consider potential covariables that might influence children’s activity patterns (e.g., physical fitness, maturation status, socioeconomic status); therefore, those variables should be considered in future studies. Fifthly, while we aimed to recruit age-matched children from Kenya and Japan, eventually, Kenyan children were significantly older than Japanese children, which might have influenced the results of the present study.

## 5. Conclusions

The study results reveal the differences in activity patterns between Kenyan and Japanese children. Overall, Kenyan children spend more time in MVPA compared to Japanese children, particularly in girls. The greatest differences in MVPA were found during weekday evenings (for both boys and girls) and weekend afternoons and evenings (for girls), suggesting that these time zones may be critical periods differentiating the overall physical activity levels between Kenyan and Japanese children. However, a significantly higher proportion of children in Japan used active transport to school compared to Kenyan children. Additionally, Japanese children spent less time in television viewing and computer gaming than Kenyan children. These findings suggest that both countries have successes and challenges that can inform effective- and country- specific intervention strategies to promote physical activity in each country.

## Figures and Tables

**Table 1:** Basic characteristics of the participants, objectively measured physical activity, and sedentary behavior.

	Boys (*n* = 139)				Girls (*n* = 159)			
	Kenya (*n* = 59)	Japan (*n* = 80)	*p*	95% CI	Kenya (*n* = 63)	Japan (*n* = 96)	*p*	95% CI
**Basic characteristics**								
Age (years)	10.9 ± 0.7	10.0 ± 0.8	<0.001	0.5, 1.0	11.0 ± 1.0	10.0 ± 0.8	<0.001	0.8, 1.3
Height (cm)	142.0 ± 11.4	133.8 ± 5.7	<0.001	6.3, 12.1	143.2 ± 8.2	132.9 ± 6.2	<0.001	8.0, 12.5
Weight (kg)	33.6 ± 6.2	31.7 ± 7.4	0.123	−0.5, 4.3	35.3 ± 6.3	30.0 ± 5.8	<0.001	3.3, 7.2
BMI	16.7 ± 4.7	17.6 ± 3.3	0.207	−2.2, 0.5	17.2 ± 2.4	16.9 ± 2.5	0.505	−0.5, 1.1
**Accelerometer data**								
**Weekday**								
SB (min/day)	518.1 ± 109.0	485.1 ± 96.0	0.206	−18.4, 84.3	566.0 ± 108.3	548.3 ± 85.9	0.428	−26.3, 61.5
LPA (min/day)	246.0 ± 31.5	293.9 ± 48.5	<0.001	−71.7, −24.0	239.1 ± 28.6	255.8 ± 38.6	0.070	−34.8, 1.36
MPA (min/day)	64.5 ± 16.7	54.5 ± 17.6	0.030	1.0, 19.2	56.9 ± 12.7	40.5 ± 12.1	<0.001	10.4, 22.3
VPA (min/day)	27.8 ± 9.1	25.5 ± 13.9	0.508	−4.6, 9.1	30.4 ± 12.0	17.0 ± 8.3	<0.001	8.9, 17.7
MVPA (min/day)	92.3 ± 24.4	80.0 ± 29.4	0.102	−2.5, 27.3	87.2 ± 21.3	57.5 ± 18.7	<0.001	20.4, 39.0
Step counts (steps/day)	14,931 ± 3208	13,214 ± 3144	0.041	72, 3362	12,499 ± 2427	10,710 ± 2088	0.001	740, 2834
Wear time (min/day)	856.4 ± 114.8	858.9 ± 92.4	0.921	−53.1, 48.0	892.3 ± 102.2	861.7 ± 93.0	0.191	−15.5, 76.7
**Weekend**								
SB (min/day)	510.5 ± 79.4	548.6 ± 148.6	0.329	−115.5, 39.3	519.1 ± 147.1	517.2 ± 108.1	0.950	−58.1, 61.9
LPA (min/day)	236.2 ± 50.4	248.1 ± 63.0	0.493	−46.1, 22.4	226.5 ± 57.1	229.9 ± 49.5	0.796	−29.6, 22.8
MPA (min/day)	50.4 ± 20.6	39.8 ± 22.8	0.100	−2.1, 23.3	43.8 ± 16.5	27.4 ± 12.5	<0.001	9.5, 23.2
VPA (min/day)	21.7 ± 11.9	18.9 ± 20.4	0.597	−7.8, 13.5	24.5 ± 11.8	12.0 ± 9.3	<0.001	7.4, 17.5
MVPA (min/day)	72.2 ± 31.1	58.7 ± 40.8	0.228	−8.6, 35.5	68.3 ± 26.3	39.5 ± 20.2	<0.001	17.8, 39.9
Step counts (steps/day)	12,131 ± 3633	11,261 ± 7665	0.662	−3090, 4831	10,158 ± 4017	8,721 ± 4289	0.192	−734, 3606
Wear time (min/day)	818.9 ± 89.1	855.4 ± 162.8	0.394	−121.5, 48.4	813.9 ± 136.2	786.6 ± 114.7	0.377	−33.8, 88.5

Data are presented as mean ±SD; BMI: body mass index; SB: sedentary behavior; LPA: light-intensity physical activity; MPA: moderate-intensity physical activity; VPA: vigorous-intensity physical activity; MVPA: moderate-to-vigorous-intensity physical activity; 95% CI: 95% confidence Interval.

**Table 2:** Domain-specific physical activity and screen time among Kenyan and Japanese children.

	Boys				Girls			
	Kenya	Japan	*p*	95% CI	Kenya	Japan	*p*	95% CI
Physical Education classes	2.92 ± 0.86	4.21 ± 0.68	<0.001	−1.60, −0.98	2.97 ± 0.90	3.97 ± 0.72	<0.001	−1.35, −0.67
Recess	3.44 ± 1.56	3.65 ± 1.36	0.690	−0.80, 0.38	3.00 ± 1.56	2.95 ± 1.31	0.910	−0.55, 0.65
Lunch	3.32 ± 1.48	3.30 ± 1.44	0.892	−0.56, 0.60	3.11 ± 1.53	2.58 ± 1.13	0.089	−0.04, 1.10
Right after school	3.56 ± 1.33	3.19 ± 1.42	0.250	−0.17, 0.92	3.36 ± 1.31	2.52 ± 1.35	0.005	0.29, 1.37
Evening	3.36 ± 1.26	3.33 ± 1.31	0.246	−0.25, 0.77	3.25 ± 1.43	2.53 ± 1.08	0.010	0.19, 1.26
Weekend	3.37 ± 1.23	2.33 ± 1.13	0.793	−0.43, 0.52	3.41 ± 1.29	2.63 ± 0.97	0.003	0.29, 1.26
After-school sport club (%)	54.2	75.6	0.009	-	50.0	43.3	0.451	-
***Weekday***								
Television time (h/day)	1.82 ± 1.67	1.66 ± 1.33	0.606	−0.45, 0.77	2.24 ± 1.75	1.42 ± 1.15	0.012	0.19, 1.45
Computer game (h/day)	1.14 ± 1.51	1.27 ± 1.29	0.646	−0.70, 0.43	1.04 ± 1.19	0.53 ± 0.82	0.021	0.08, 0.95
***Weekend***								
Television time (h/day)	3.81 ± 1.58	2.49 ± 1.53	<0.001	0.69, 1.94	3.23 ± 1.76	2.17 ± 1.34	0.002	0.40, 1.71
Computer game (h/day)	2.03 ± 1.83	1.79 ± 1.49	0.471	−0.43, 0.93	1.68 ± 1.82	0.84 ± 1.11	0.012	0.19, 1.41

Physical activity scores were evaluated using the Physical Activity Questionnaire for Children (PAQ-C) [24,25]. Higher physical activity scores indicate higher physical activity levels; 95% CI: 95% confidence interval.
